# Influence of Additives on the Macroscopic Color and Corrosion Resistance of 6061 Aluminum Alloy Micro-Arc Oxidation Coatings

**DOI:** 10.3390/ma17112621

**Published:** 2024-05-29

**Authors:** Xuanyu Chen, Hao Wang, Cancan Liu, Wenqiang Wang, Bo Chen

**Affiliations:** College of Materials Science and Engineering, Nanjing Tech University, Nanjing 211800, China; 202161203207@njtech.edu.cn (X.C.); 202261203312@njtech.edu.cn (H.W.); 202161203202@njtech.edu.cn (W.W.); 202161203196@njtech.edu.cn (B.C.)

**Keywords:** aluminum alloy, white micro-arc oxidation coating, decorative, corrosion resistance

## Abstract

In this study, we successfully employed the plasma electrolytic oxidation (PEO) technique to create a uniform white ceramic layer on the surface of the 6061 aluminum alloy using K_2_ZrF_6_ and Na_2_WO_4_ as colorants. A scanning electron microscope (SEM) equipped with an energy-dispersive X-ray spectrometer (EDS) and X-ray diffraction (XRD) were used to characterize the coatings, and we used an electrochemical workstation to test their corrosion protection properties. The corrosion resistance of the coatings was analyzed using potentiodynamic polarization curves. The results showed that K_2_ZrF_6_ addition whitened the coating with ZrO_2_ as the main phase composition, inhibiting Al substrate depletion and enhancing coating corrosion resistance. A small amount of Na_2_WO_4_ decreased the coating’s L* value, successfully constructing ceramic coatings with L* (coating brightness) values ranging from 70 to 86, offering broad application prospects for decorative coatings.

## 1. Introduction

In recent years, aluminum and its alloys have been extensively utilized in various fields, such as aerospace and 3C electronics due to their high specific strength, excellent corrosion resistance, and good thermal conductivity [1,2,3,4]. However, certain products, particularly electronic devices, necessitate surface coloring treatment to meet decorative requirements. Common coloring techniques for aluminum alloys, including dye coloring and anodic oxidation coloring, are limited by factors such as low hardness, poor bonding, and complex processes [5,6,7].

PEO technology, a novel surface treatment approach evolved from traditional anodic oxidation technology, has significantly enhanced processing efficiency by increasing the anode voltage from tens to hundreds of volts. In this process, valve metals (e.g., Mg, Al, and Ti) and their alloys are placed in an alkaline electrolyte as the anode, while stainless steel and other inert electrode plates serve as the cathode. When the voltage surpasses the metal anode’s capacitance threshold, plasma discharge occurs, and a ceramic layer grows on the metal substrate surface. This coating exhibits properties such as high bonding strength and good corrosion resistance [8,9]. Moreover, the alkaline electrolyte is easier to dispose of, reducing environmental pollution. By introducing different additives to the electrolyte, coatings of various colors can be prepared on the metal surface. Indeed, black, white, green, blue, and other colored coatings have been successfully produced on aluminum alloys [10,11]. The commonly used colorants include Na_2_WO_4_, K_2_ZrF_6_, NH_4_VO_3_, K_2_TiF_6_, etc. [12,13,14]. Wang et al. [15] prepared white, brown, grey, and black coatings on the surface of the AZ91D magnesium alloy using copper acetate, sodium tungstate, and sodium orthovanadate. It was not difficult to find that the presentation of different colors was caused by the formation of oxides on the coating surface by different metal cations. When the AZ31 magnesium alloy is micro-arc-oxidized in a cerium nitrate electrolyte, the coating shows a yellow color due to the combined effect of cerium dioxide and cerium oxide, and prolonging the time of micro-arc oxidation can make the yellow color deepen [16]. Jiang [17] varied the amount of sodium hexametaphosphate to investigate its effect on the color of the coating. However, these coating colors were only qualitative, lacking quantitative data expression, such as color interval ranges. Although numerous studies have investigated colored coatings, few have focused on different shades of the same coating color. This study aimed to adjust the coating color using different colorants, establish a library of white color gradients for the coating, and investigate the coating’s corrosion resistance, thereby extending the application field of PEO technology.

In this study, Na_2_WO_4_ and K_2_ZrF_6_ were selected as colorants to prepare white coatings on aluminum alloy surfaces using PEO. By varying the colorant content, the coatings’ chromaticity value, L* [18] (brightness), was made to fall within the range of 70–86, making them adaptable for decorative purposes in different fields. The corrosion resistance and coloring mechanism of the coatings were also analyzed and discussed.

## 2. Experimental Procedures

### 2.1. Coating Deposition Conditions

The substrate used was a 6061 aluminum alloy with dimensions of Φ35 × 4 mm (wt%: Cu 0.28, Mg 0.88, Fe 0.34, Si 0.5, Mn 0.14, Zn 0.09, Cr 0.12, Ti 0.4, and Al). Each sample’s surface was polished with 2000 mesh silicon carbide paper and ultrasonically cleaned in ethanol. All chemicals used in the coating preparation process were analytically pure (AR), with specific details listed in Table 1.

Table 2 presents the electrolyte compositions and denotes the samples as P-Zr0, P-Zr2, P-Zr4, P-Zr15, P_6_-W0, P_6_-W0.4, and P_6_-W0.8. PEO was performed using a pulsed unipolar power supply (10 W) in the constant current mode, with the solution temperature maintained below 20 °C through cooling and stirring. The micro-arc oxidation process parameters were set to a current density of 2 A/dm^2^, a duty cycle of 20%, a frequency of 500 HZ, and a treatment time of 15 min.

### 2.2. Microscopic Characterization

The PEO coatings’ morphology and surface elemental composition were observed using scanning electron microscopy (SEM, JEOL, JSM-7900F, Tokyo, Japan) and energy dispersive X-ray spectroscopy (EDS, JEOL, JSM-IT500A, Japan). In the SEM technique, the accelerating voltage was 15 KV, with the backscattering mode used for the coating cross-section scans, and the secondary electron mode was used for the coating surface scans. X-ray diffraction (XRD, D/Max-2400, Akishima, Japan) was employed to determine the coatings’ phase composition using a Cu target (Kα1 = 0.15406 nm) as the anode target in the parallel light mode with a grazing angle of 2°. The samples were scanned in the 2θ range from 20 to 90° with a step size of 0.02° and a scanning speed of 10°/min. An electrochemical workstation (Metrohm Autolab PGSTAT302 N, Herissau, Switzerland) was used to test the coatings’ corrosion protection properties in a 3.5 wt.% NaCl solution at room temperature, employing a conventional three-electrode battery system with a Ag/AgCl electrode as the reference electrode and a platinum electrode as the counter electrode. Potentiodynamic polarization curves were tested at a scan rate of 10 mV/s after 1 h of immersion. The coating thickness was measured using an eddy current thickness gauge (FMP20, Fisher, Schwerte, Germany), while optical emission spectroscopy (OES, Ideaoptics PG2000-Pro, Shanghai, China) was used to study the discharge sparks’ emission spectra during the PEO process. The surface roughness was measured using a surface roughness tester (Ra200, Jingmere Technology Co., Ltd., Beijing, China). The coatings’ chromaticity was analyzed using the LS171 colorimeter to obtain the chromaticity values L* (luminance), a* (red to green range), and b* (yellow to blue range).

## 3. Results and Discussion

### 3.1. Voltage–Time Response

**Figure 1:**
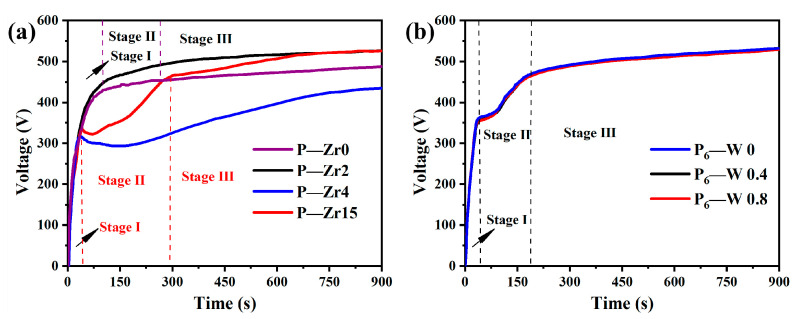
Time–voltage response curve of the aluminum alloy micro-arc oxidation process: (**a**) after adding K_2_ZrF_6_; (**b**) after adding Na_2_WO_4_.

The time–voltage curve for the micro-arc oxidation process after adding K_2_ZrF_6_ is shown in Figure 1a. The three stages of micro-arc oxidation are delineated by dashed lines. When the content of K_2_ZrF_6_ is between 0 g/L and 2 g/L, the electrolyte remains stable. However, when the content of K_2_ZrF_6_ is increased to 4 g/L and 15 g/L, a significant voltage drop occurs during the first stage of micro-arc oxidation, known as the anodic oxidation stage. This is due to the generation of a large number of bubbles and the formation of the oxide layer. As the working voltage reaches the breakdown voltage, the process enters the second stage of micro-arc oxidation. At this point, the acidic solution partially dissolves the newly formed oxide layer, creating pores and resulting in a decrease in the coating resistance, leading to a voltage drop. As the reaction progresses and the coating growth rate surpasses the dissolution rate, the working voltage gradually rises and stabilizes.

Figure 1b illustrates the time–voltage curve after adding a small amount of Na_2_WO_4_ solution. Due to the low Na_2_WO_4_ content, the solution stabilizes, and the time–voltage curves of the three electrolytes are consistent. When the voltage reaches the breakdown voltage, entering the second stage of micro-arc oxidation, the rate of voltage rise is lower than in the first stage, which is also attributed to the acidic nature of the solution.

### 3.2. Effect of Different Additives on the Surface Structure and Micro-Structure of the Coatings

**Figure 2:**
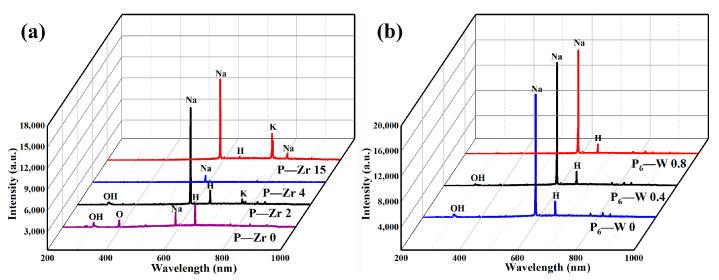
OES spectra of aluminum alloy micro-arc oxidation at the same time: (**a**) in the K_2_ZrF_6_ solution with different contents; (**b**) in the Na_2_WO_4_ solution with different contents.

Based on the micro-arc oxidation time–voltage curve, a point was selected at the third stage of micro-arc oxidation (300th s of the large spark stage). The discharged sparks generated during the process were captured, and the discharged ions were analyzed using optical emission spectroscopy (OES) between 200 and 1000 nm. Figure 2a shows the OES spectra of the four solutions after adding K_2_ZrF_6_. The discharge intensity of the electrolyte solution first increases, then decreases, and finally increases with the addition of different K_2_ZrF_6_ content, consistent with the trend of its operating voltage. Comparing the solutions with and without K_2_ZrF_6_ reveals that during discharging, not only the strong discharge of Na 589.46 nm is present, but also K 767.11 nm is involved in the discharge reaction. This indicates that the electrolyte is primarily consumed during the discharge process. The absence of an Al peak in the spectra further suggests that the substrate is seldom consumed in the energy supply discharge. Additionally, Hα 656.41 nm in the electrolyte also participates in the reaction, which is related to the acidic nature of the solution after the hydrolysis of K_2_ZrF_6_. Figure 2b shows the OES spectra after adding a small amount of Na_2_WO_4_. The spectra stabilize due to the low Na_2_WO_4_ content and the lack of obvious voltage changes during the discharge process. The presence of strong Na peaks further indicates that the discharge process mainly consumes the electrolyte.

**Figure 3:**
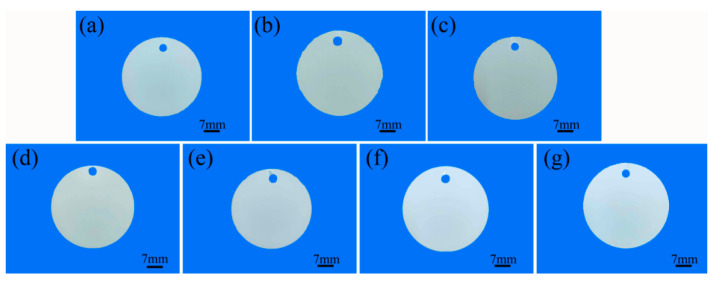
Comparison of the macroscopic morphologies of the aluminum alloy micro-arc oxidation coatings with different additives: (**a**) P6—W 0, (**b**) P6—W 0.4, (**c**) P6—W 0.8, (**d**) P—Zr 0, (**e**) P—Zr 2, (**f**) P—Zr 4, and (**g**) P—Zr 15.

Figure 3 illustrates the macroscopic color of the micro-arc oxidation coating after adding additives to the two electrolyte systems. The coating color becomes darker with the addition of a small amount of Na_2_WO_4_, while the addition of K_2_ZrF_6_ results in a whiter coating due to the presence of the corresponding tungsten and zirconium compounds. Tungstate is commonly used in the preparation of black coatings for micro-arc oxidation, so even a small amount of Na_2_WO_4_ can significantly decrease the whiteness. ZrO_2_, being a white compound, further demonstrates that the formation of micro-arc oxide coatings involves the deposition of compounds from the electrolyte, as evidenced by the change in coating color.

To express the difference in the coating color visually and accurately, a color difference analyzer was employed to demonstrate the coating color change through CIE L* a* b*. As shown in Table 3, with an increasing K_2_ZrF_6_ concentration, the coating color gradually becomes lighter and tends to be pure. Tu et al. found that K_2_ZrF_6_ does not continuously enhance the whiteness of the coating, and when the concentration reaches a certain level, the whiteness rises slowly [18]. Simultaneously, the addition of a small amount of Na_2_WO_4_ darkened the coating color, with the L* value dropping as low as 70. Table 4 presents the surface element contents of the aluminum alloy micro-arc oxidation coatings with different additives. Before adding the additives, the main elements of the coating were Al and O, and the coating color was influenced by the substrate composition. After adding K_2_ZrF_6_, the coating elements primarily consisted of Al, O, and Zr. The Zr in the coating originated from the electrolyte and increased with the rise in K_2_ZrF_6_ content, indicating that the electrolyte composition plays a major role in the coating growth process. The decrease in the Al content also suggests that the addition of K_2_ZrF_6_ suppresses the influence of the Al substrate on the coating color, ultimately leading to an increase in the coating L* value. Similarly, W was also present in the dark coating, but the small amount of Na_2_WO_4_ content resulted in a low W content in the coating. However, even a small amount of W led to a deeper coating color, as Na_2_WO_4_ is commonly used in the preparation of black coatings.

**Figure 4:**
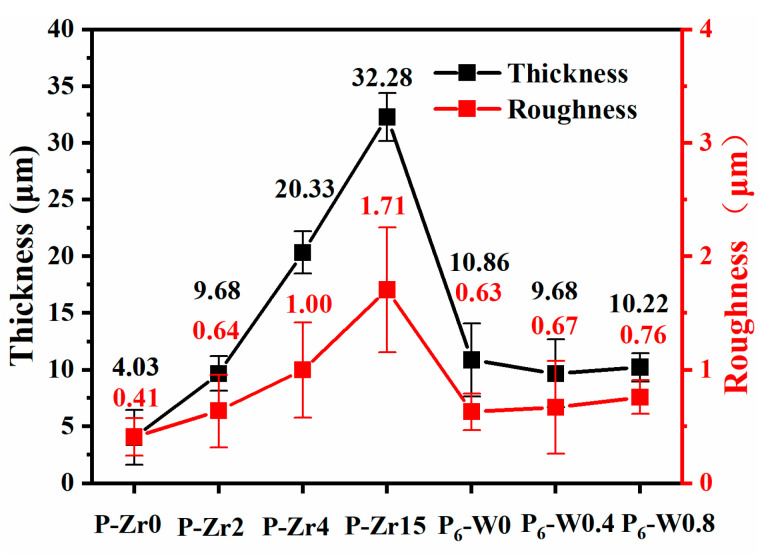
Relationship between the thickness and surface roughness of the aluminum alloy micro-arc oxidation coatings with different additives.

Figure 4 illustrates the relationship between the thickness and roughness of the aluminum alloy micro-arc oxidation coatings with different additives. It was observed that with an increasing K_2_ZrF_6_ concentration, the thickness of the micro-arc oxidation coating under the same electric parameters increased, indicating that zirconium ions participated in the reaction and were incorporated into the coating. K_2_ZrF_6_ fully dissolved, further suggesting that K_2_ZrF_6_ promotes coating growth. The increase in thickness also led to an increase in coating roughness, and the changing trend of roughness was consistent with the changing trend of thickness, as evident in the figure. This demonstrates that within the same electrolyte, the coating thickness is a direct factor affecting the surface roughness of the coating. Simultaneously, due to the low Na_2_WO_4_ content and the same working voltage, no obvious changes in coating thickness or roughness were observed.

**Figure 5:**
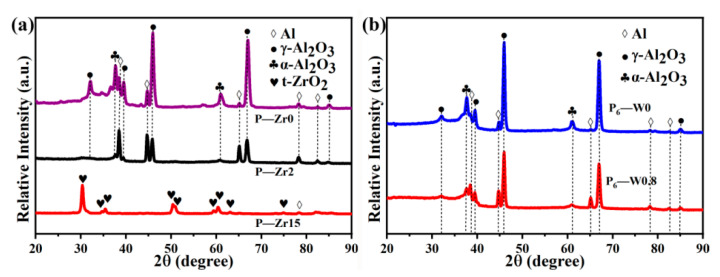
XRD patterns of micro-arc oxidation coatings on aluminum alloys (**a**) with different contents of K_2_ZrF_6_ and (**b**) with different contents of Na_2_WO_4_.

Figure 5 shows the results of the physical phase analysis of the micro-arc oxidation coatings prepared with different additives. From Figure 5a, it can be seen that the main phase compositions of the coatings without the addition of K_2_ZrF_6_ are α-Al_2_O_3_ and γ-Al_2_O_3_, with a small amount of the Al substrate diffraction peaks also present. This is because the micro-arc oxidation coating has a loose, porous structure, and the X-rays pass through the pores on the coating surface, revealing the diffraction peaks of the Al substrate. With the addition of K_2_ZrF_6_, a gradual decrease in α-Al_2_O_3_ and γ-Al_2_O_3_ is clearly observed, indicating that the addition of K_2_ZrF_6_ reduces the consumption of the Al substrate. When the K_2_ZrF_6_ content reached 15 g/L, α-Al_2_O_3_ and γ-Al_2_O_3_ completely disappeared, as the increase in the K_2_ZrF_6_ content completely suppressed the depletion of the Al substrate. All the Zr in the coatings came from the electrolyte, and tetragonal zirconia (t-ZrO_2_) appeared, which was the reason for the increase in the L* values of the coatings. This further demonstrates that the presence of additives in the micro-arc oxidation process inhibits substrate depletion, and the coating growth is mainly derived from the deposition of electrolyte compounds, with the coating growth mode dominated by deposition. Moreover, the thickening of the coating has a shielding effect, which reduces the Al diffraction peaks. Figure 5b shows the change in the coating phase composition after adding Na_2_WO_4_. Due to the small content of the additive, Na_2_WO_4_ could not affect the coating phase composition, and the coating maintained α-Al_2_O_3_ and γ-Al_2_O_3_ as the main crystalline phases.

**Figure 6:**
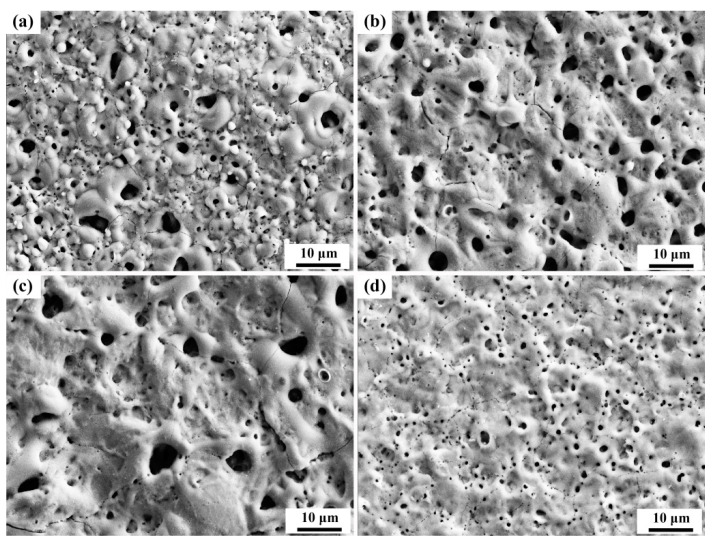
Surface morphology of the aluminum alloy micro-arc oxidation coatings (15 min) with different K_2_ZrF_6_ contents: (**a**) P—Zr 2, (**b**) P—Zr 4, (**c**) P—Zr 15, (**d**) P—Zr 0.

**Figure 7:**
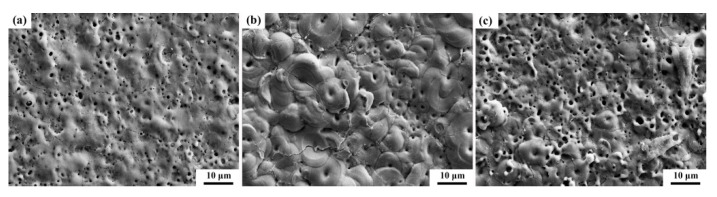
Surface morphology of the aluminum alloy micro-arc oxidation coatings (15 min) with different Na_2_WO_4_ contents: (**a**) P6—W 0.4, (**b**) P6—W 0.8, (**c**) P6—W 0.

Figure 6 and Figure 7 illustrate the surface morphology of the coatings with the two additives. The images reveal that the coating surfaces generated after the micro-arc oxidation treatment exhibited more discharge channels and cracks, which were caused by excessive cooling contraction, leading to brittle fractures within the internal texture. Figure 6d shows the surface morphology of the coatings without the addition of K_2_ZrF_6_, characterized by numerous small holes and the absence of the “pancake” structure typically observed after strong B-type discharge in the base electrolyte [19]. This phenomenon is attributed to the acidic nature of the electrolyte, where the coating growth rate is lower than the dissolution rate, and both rates remain consistent, thereby explaining the lack of a decrease in the operating voltage. However, with the addition of K_2_ZrF_6_, as depicted in Figure 6a, a granular structure emerged in the coating due to the involvement of K_2_ZrF_6_ in the reaction, with discharges occurring in the form of A and C-type discharges. Previous studies have reported that A and C-type discharges tend to incorporate electrolyte substances into the coating, while B-type discharges dope the coating through the melting of the substrate components. The growth of the coating during micro-arc oxidation is attributed to the oxidation of molten aluminum as it exits through the discharge channel created by the breakdown of the oxide layer [20]. Consequently, the formed aluminum oxide is ejected from the channel, encountering the rapidly cooled electrolyte on the coating surface, forming a pancake structure and indicating that the main component of the film originates from the substrate metal, highlighting the significance of B-type discharges. In contrast, both A and C-type discharges occur in the upper layer of the coating without contacting the substrate, involving only the interaction between the electrolyte and the oxide in the coating. As the K_2_ZrF_6_ content increased, the alumina content in the coating decreased until it disappeared, explaining the absence of the granular structure in Figure 6c. It was replaced by fewer and larger pores, which provided the foundation for the thermal control properties of the coating. Figure 7b displays a distinct “pancake” structure after B-type discharge. The surface morphology in Figure 7a resembles that in Figure 6d, which is attributed to the acidity of the solution. Although the additives induced A and C-type discharges, the discharges still occurred in the form of B-type discharges due to the low content of Na_2_WO_4_.

**Figure 8:**
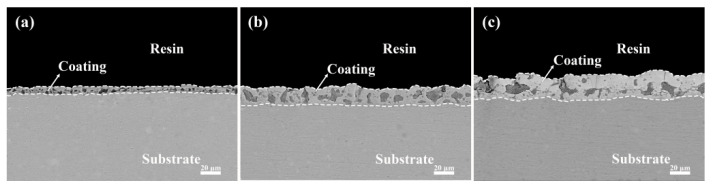
Cross-sectional morphology of the aluminum alloy micro-arc oxidation coatings (15 min) with different K_2_ZrF_6_ contents: (**a**) P—Zr 2, (**b**) P—Zr 4, (**c**) P—Zr 15.

**Figure 9:**
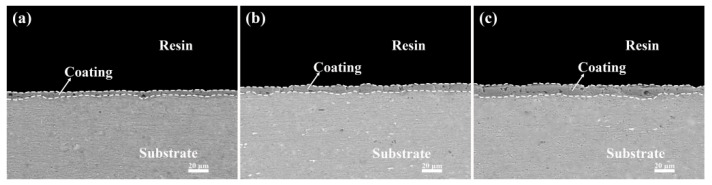
Cross-sectional morphology of the aluminum alloy micro-arc oxidation coatings (15 min) with different Na_2_WO_4_ contents: (**a**) P6—W 0, (**b**) P6—W 0.4, (**c**) P6—W 0.8.

The cross-sectional morphology of the six coatings is presented in Figure 8 and Figure 9. It is observed that the cross-sections of the micro-arc oxidation coatings after the addition of K_2_ZrF_6_ exhibit similar characteristics, all featuring the presence of macropores. The varying contents of K_2_ZrF_6_ resulted in different coating thicknesses, which is consistent with the findings in Figure 4. This further confirms that the addition of K_2_ZrF_6_ promotes coating growth, and the decrease in the working voltage does not impact the coating thickness. Moreover, as the K_2_ZrF_6_ concentration increased, the zirconium-containing oxides filled the coating pores more uniformly, attributable to the fact that the product of K_2_ZrF_6_ after electrolysis facilitated the formation and buildup of the coating during the micro-arc oxidation process. The coating with the addition of Na_2_WO_4_ did not exhibit significant changes, except for the thickness due to its small amount.

**Figure 10:**
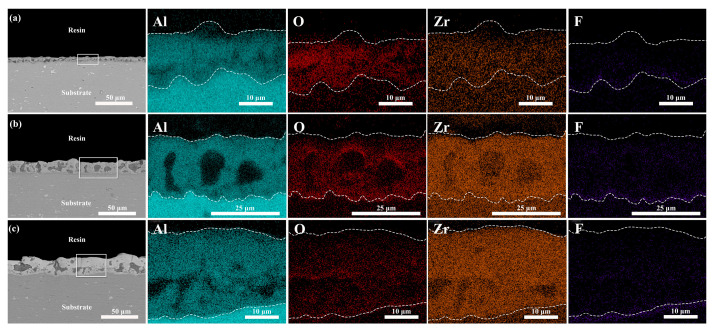
Cross-sectional elemental distribution of the aluminum alloy micro-arc oxidation coatings after 15 min at different K_2_ZrF_6_ contents: (**a**) P—Zr 2, (**b**) P—Zr 4, (**c**) P—Zr 15.

**Figure 11:**
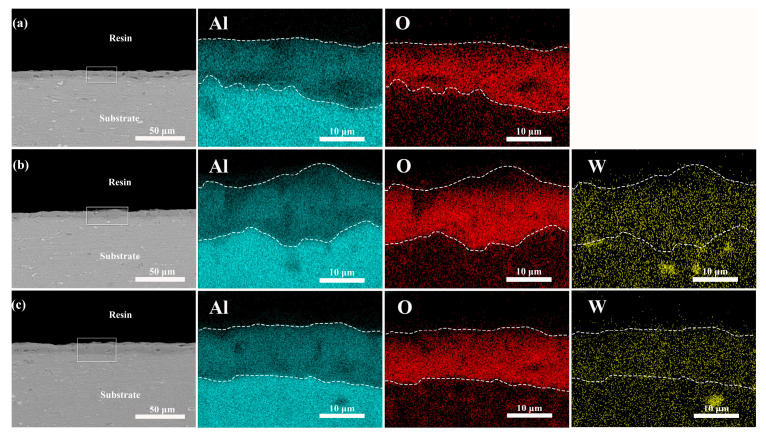
Cross-sectional elemental distribution of the aluminum alloy micro-arc oxidation coatings after 15 min at different Na_2_WO_4_ contents: (**a**) P6—W 0, (**b**) P6—W 0.4, (**c**) P6—W 0.8.

Table 4 reveals that in the absence of K_2_ZrF_6_, the coating is predominantly composed of Al and O, indicating that alumina is the primary constituent. Figure 10 and Figure 11 illustrate the elemental distribution of the coatings with the addition of K_2_ZrF_6_ and Na_2_WO_4_, respectively. It is observed that after the addition of K_2_ZrF_6_, the coating is primarily composed of Al, O, and Zr, with a significant increase in the Zr content as the K_2_ZrF_6_ content increases. The XRD pattern suggests that the coating is dominated by ZrO_2_ at this point, which increases with the rise in K_2_ZrF_6_ content. Furthermore, the shallow distribution of Al elements in the coating indicates that the addition of K_2_ZrF_6_ inhibits the consumption of the Al substrate, aligning with the XRD results. The F element did not exhibit a significant presence, potentially due to the short oxidation time. According to Tu’s study, F was found to enter the coating at the late stage of micro-arc oxidation [21]. The coating without Na_2_WO_4_ and the coating without K_2_ZrF_6_ share the same main elemental composition of alumina. After the addition of Na_2_WO_4_, W is uniformly distributed in the coating, confirming that a small amount of Na_2_WO_4_ participates in the micro-arc oxidation process and enters the coating, deepening its color. However, due to the small quantity of Na_2_WO_4_, no crystalline phase is observed.

### 3.3. Effect of Various Additives on the Corrosion Resistance of Aluminum Alloy Coatings

**Figure 12:**
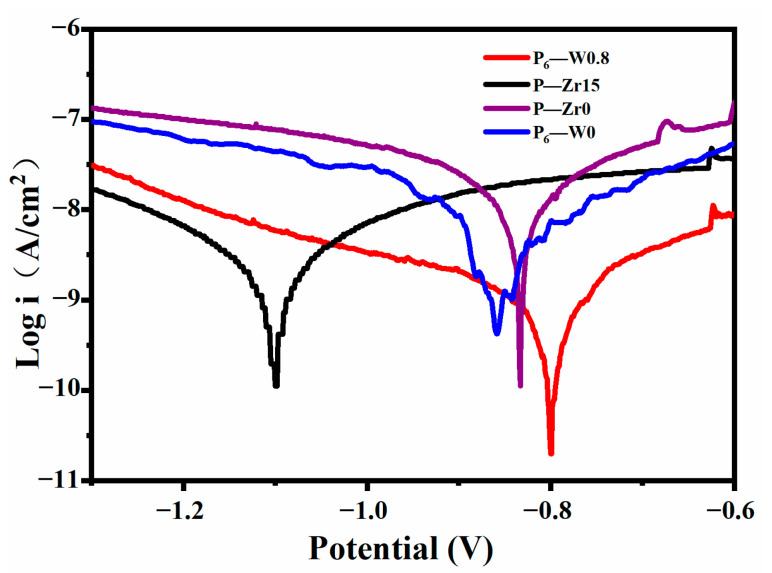
Kinetic potential polarization curves of the aluminum alloy micro-arc oxidation coatings with different additives immersed in a 3.5 wt.% NaCl solution for 1 h.

Figure 12 presents the kinetic potential polarization curves measured in a 3.5% NaCl solution at room temperature for samples with and without additives after micro-arc oxidation. Table 5 lists the fitting results of the corrosion potential (Ecorr) and corrosion current density (icorr). In general, the corrosion potential reflects the coating’s stability; a more positive corrosion potential indicates a more stable coating. The corrosion current density reflects the coating’s corrosion rate; a lower corrosion current density corresponds to a more corrosion-resistant coating. The Ecorr is typically related to the material’s thermodynamic stability, and a lower Ecorr value suggests a higher susceptibility to localized corrosion [22]. The addition of Na_2_WO_4_ resulted in a 53 mV increase in the Ecorr compared to P6-W0, which may be attributed to the increased crystallinity of the coating. Furthermore, the icorr value decreased after the addition of Na_2_WO_4_, indicating the improved corrosion resistance of the coatings. The addition of K_2_ZrF_6_ also led to a one-order-of-magnitude decrease in the corrosion current density (icorr), which is due to the increased coating thickness and the consequent slowing of the corrosion rate, resulting in better corrosion protection performance. However, the corrosion potential (Ecorr) decreased by 268 mV, and the cross-sectional morphology of the coatings revealed a loose and porous structure after the addition of K_2_ZrF_6_, making them more susceptible to corrosion. The increased thickness contributed to the enhanced corrosion resistance of the coating in the same environment [23].

### 3.4. Coloring Analysis

The presence of ZrO_2_ on the surface is the primary reason for the coating’s white color. The additives in the electrolyte during the oxidation process led to the occurrence of A and C-type discharges on the coating surface. These additives are doped into the porous layer and inhibit the generation of Al_2_O_3_. The elemental distribution of the coating cross-section further confirms the deposition of colorants within the layer. Although a small amount of Na_2_WO_4_ may not produce the crystalline phase of WO_2_, the presence of the W element contributes to the darkening of the coating color. This explains the observed change in the coating color from darker to lighter colors.

### 3.5. Limitations and Future Research

Despite the classification of coating chromaticity under the same color system using the additives, the color range is relatively limited. Therefore, this system can be expanded and enriched. Moreover, the content of additives depends on the final rendering of the coating, requiring more pre-experiments for verification.

Cost reduction can be achieved not only by adjusting the additives but also by modifying the electrical parameters, such as the oxidation time and current density. To achieve coating color diversification, substrates with trace color elements can be employed in experiments.

Furthermore, the addition of K_2_ZrF_6_ results in a coating that not only meets decorative requirements but also exhibits a low absorption rate. The presence of zirconium oxide in the coating improves its emissivity, making it suitable for aerospace applications.

## 4. Conclusions

Ceramic layers with L* values between 70 and 86 were successfully prepared using PEO technology. The following conclusions can be drawn from the characterization and corrosion resistance studies of the coatings prepared under different conditions:

1. K_2_ZrF_6_ and Na_2_WO_4_ were fully dissolved and involved in the reaction, resulting in micro-arc oxidation coatings with L* values between 70 and 86 after adding the additives. A crystalline phase of ZrO_2_ appeared in the coating, which lightened the coating color, while a small amount of Na_2_WO_4_ did not form a crystalline phase, but the presence of elemental W in the coating darkened the coating color;
2. The addition of additives to the coating during the reaction decreased its thermal stability due to the reduction of the crystalline phase compared to the coatings without the additives. However, the corrosion current decreased by one order of magnitude, indicating improved corrosion resistance. The addition of the additives not only enhanced the corrosion resistance of the coating but also increased its wear resistance, extending the service life of the aluminum alloy;
3. The addition of additives inhibited the depletion of the Al substrate, and the crystalline phases α-Al_2_O_3_ and γ-Al_2_O_3_ in the coatings decreased with an increasing additive concentration. Moreover, the addition of the additives altered the discharge type, leading to changes in the coating surface morphology. The increased coating thickness and the presence of macropores in the K_2_ZrF_6_ solution laid the foundation for the thermal control performance of the coatings.

## Figures and Tables

**Table 1:** List of the chemical reagents used in the experiment.

Drug Name	Chemical Formula	Manufacturer
Trisodium phosphate	Na₃PO₄	Sinopharm Chemical Reagent Co. (Shanghai, China)
Sodium silicate	Na_2_SiO_3_	Sinopharm Chemical Reagent Co. (Shanghai, China)
Sodium Hexametaphosphate	(NaPO_3_)_6_	Anhui Zhongxu Biotechnology Co. (Anhui, China)
Sodium tungstate	Na_2_WO₄	Xilong Science Co. (Guangzhou, China)
Potassium Hexafluorozirconate	K_2_ZrF_6_	Shanghai Aladdin Biochemical Technology Co. (Shanghai, China)

**Table 2:** Composition of the seven electrolytes.

Specimen	Electrolyte	pH	Conductivity (mS/cm)
P-Zr0	18 g/L Na_3_PO_4_ + 3 g/L Na_2_SiO_3_ + Corrosion inhibitor A	5.34	10.41
P-Zr2	18 g/L Na_3_PO_4_ + 3 g/L Na_2_SiO_3_ + 2 g/L K_2_ZrF_6_ + Corrosion inhibitor A	5.40	11.15
P-Zr4	18 g/L Na_3_PO_4_ + 3 g/L Na_2_SiO_3_ + 4 g/L K_2_ZrF_6_ + Corrosion inhibitor A	5.28	11.80
P-Zr15	18 g/L Na_3_PO_4_ + 3 g/L Na_2_SiO_3_ + 15 g/L K_2_ZrF_6_ + Corrosion inhibitor A	5.35	15.75
P_6_-W0	35 g/L (NaPO_3_)_6_ + 5 g/L Na_2_SiO_3_	6.77	11.88
P_6_-W0.4	35 g/L (NaPO_3_)_6_ + 5 g/L Na_2_SiO_3_ + 0.4 g/L Na_2_WO_4_	6.42	12.46
P_6_-W0.8	35 g/L (NaPO_3_)_6_ + 5 g/L Na_2_SiO_3_ + 0.8 g/L Na_2_WO_4_	6.25	12.88

**Table 3:** L* a* b* CIE results of the aluminum alloy micro-arc oxidation coatings with different additives.

Specimen	L*	a*	b*
P-Zr0	70.23	1.44	5.57
P-Zr2	77.15	1.01	1.74
P-Zr4	82.17	0.75	0.51
P-Zr15	85.33	0.80	0.37
P_6_-W0	75.18	0.76	2.92
P_6_-W0.4	72.13	1.03	3.11
P_6_-W0.8	69.67	1.42	5.86

**Table 4:** Surface element content of the aluminum alloy micro-arc oxidation coatings with different additives.

Specimen	Content of Elements (wt.%)			
	O	Al	Zr	W
P-Zr0	53.20	46.80	--	--
P-Zr2	47.92	42.45	9.63	--
P-Zr4	37.58	37.43	24.99	--
P-Zr15	27.36	17.39	55.25	--
P_6_-W0	55.37	44.63	--	--
P_6_-W0.4	49.80	46.79	--	3.41
P_6_-W0.8	48.66	44.73	--	6.61

**Table 5:** Fitting results of the dynamic potential polarization curves.

Specimen	*E_corr_* (mV vs. Ag/AgCl)	*i_corr_* (A/cm^2^)
P_6_-W0	−854	5.032 × 10^−9^
P_6_-W0.8	−801	8.960 × 10^−10^
P-Zr0	−832	1.064 × 10^−8^
P-Zr15	−1100	1.732 × 10^−9^

## Data Availability

Data are contained within the article.
